# Genetic Immunisation by Liver Stage Antigen 3 Protects Chimpanzees against Malaria despite Low Immune Responses

**DOI:** 10.1371/journal.pone.0002659

**Published:** 2008-07-16

**Authors:** Pierre Daubersies, Benjamin Ollomo, Jean-Pierre Sauzet, Karima Brahimi, Blanca-Liliana Perlaza, Wijnand Eling, Hubert Moukana, Pierre Rouquet, Charles de Taisne, Pierre Druilhe

**Affiliations:** 1 Unité de Parasitologie Biomédicale, Institut Pasteur, Paris, France; 2 Centre International de Recherches Médicales de Franceville, Franceville, Gabon; 3 Department of Medical Microbiology, University of Nijmegen, Nijmegen, The Netherlands; 4 Aventis-Pasteur, Lyon, France; Instituto de Pesquisa Clinica Evandro Chagas, FIOCRUZ, Brazil

## Abstract

**Background:**

The true interest of genetic immunisation might have been hastily underestimated based on overall immunogenicity data in humans and lack of parallelism with other, more classical immunisation methods.

**Principal Findings:**

Using malaria Liver Stage Antigen-3 (LSA-3), we report that genetic immunization induces in chimpanzees, the closest relative of humans, immune responses which are as scarce as those reported using other DNA vaccines in humans, but which nonetheless confer strong, sterile and reproducible protection. The pattern was consistent in 3/4 immunized apes against two high dose sporozoite challenges performed as late as 98 and 238 days post-immunization and by a heterologous strain.

**Conclusions:**

These results should, in our opinion, lead to a revisiting of the value of this unusual means of immunisation, using as a model a disease, malaria, in which virulent challenges of volunteers are ethically acceptable.

## Introduction

At its advent, the use of genetic immunisation generated considerable expectations. The initial enthusiasm was based on the ease of production and purification of the immunogens as compared to proteins, the simplicity of the approach and the high immunogenicity in all animal models, including primates employed at the pre-clinical level. This was followed by a period of disillusionment and decreased interest, in particular from the industrial sector, when it became clear that, for reasons that remain poorly understood, the same high immunogenicity could not be reproduced in humans. Genetic immunisation of volunteers could induce at best CTL cells but low, or absent, CD4 T-cell and antibody responses. The immunogenicity was so low that, although reported, several clinical trials were considered unsuitable for publication. In many of these trials, challenge by the infectious pathogens was either not possible or decided against in view of the limited responses induced.

In the past, we have employed the chimpanzee as a model system to study malaria because it is receptive to challenge by the same species that infect humans, shares a very high degree of homology with human beings (99.4%), has immune responses that closely mimic those obtained in humans and can be immunologically investigated in nearly as much detail as mice and humans. We have previously reported that protection could be obtained in chimpanzees using Liver Stage Antigen-3, a new *P. falciparum* protein identified by differential screening of immune responses from protected and non-protected volunteers similarly immunized with the irradiated parasite, which is expressed in sporozoite and Liver stages [1]. In contrast to many other vaccine candidates, the regions of immunological relevance are highly conserved. Protection has been demonstrated in the chimpanzee, both by lipopeptide immunization without adjuvant as well as by recombinant protein immunization with an adjuvant acceptable for human use, and in *Aotus* monkeys using non-adjuvated particulate formulations [2].

Since the simplicity and ease of genetic immunization makes it a valuable tool for the large scale Public Health problem posed by malaria [3], [4] we also wished to investigate the potential of LSA-3 presentation via naked DNA. The LSA-3 DNA vaccine, denoted VR-LSA3, consisted of the pVR1020 plasmid into which a PCR-amplified fragment coding for the largest part of the *lsa-3* gene from *P. falciparum* (the most hydrophobic C-terminal part was deliberately deleted) was cloned in frame within the expression cassette. We first exploited the homology between the *P. falciparum* and *P. yoelii* LSA-3 antigens [5]. The *P. falciparum* LSA-3 construct was used to demonstrate that genetic immunization of mice could induce substantial protection against sporozoite challenge by the heterologous species *P. yoelii* [5], [6], [7]. These results, together with the strong protection obtained previously using sub-unit LSA-3 formulations, led us to initiate genetic vaccination and challenge studies in higher primates with *P. falciparum* LSA-3 in Vical vector. Previous studies have shown that following *P. falciparum* sporozoite challenges in the chimpanzee the reproducibility of blood infections was remarkable [1] Although a limited number of animals can be enrolled, the clear differences between vaccines and controls, and the possibility to repeat challenges, provide significant data. This was particularly well demonstrated in animals undergoing five successive challenges [1].

## Results and Discussion

**Figure 1:**
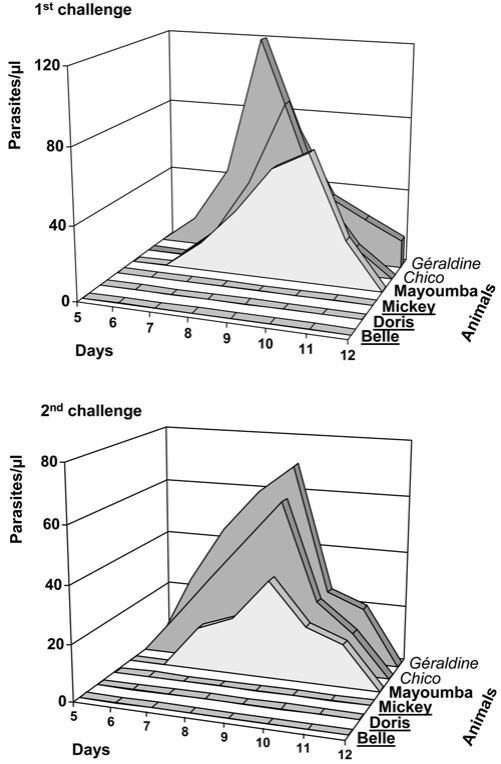
Blood parasitaemia profiles in the chimpanzees after both sporozoite challenges. Names of control animals are italicized. Names of protected LSA-3 immunized chimpanzees are underlined. All results were confirmed by PCR and QBC analysis of the daily blood samples.

Six adult chimpanzees were included in the study[8], [9]: four were injected three times at 4–5 week intervals with a one mg dose of VR-LSA3, and two control animals were similarly immunized with a control plasmid coding for a non-malaria related antigen, the Respiratory Syncitial Virus protein. During and following the immunization period, no adverse local or systemic reactions were observed. A first challenge was performed 14 weeks after the third immunization, by intravenous injection of 20,000 sporozoites from the *P. falciparum* NF54 strain. In control animals, blood stage parasites were detectable as soon as day six or seven post-challenge, i.e. reflecting the first invasion of red blood cells by the liver merozoites emerging from hepatic schizonts (Fig. 1). Three of the four immunized animals were fully protected - no parasitized red blood cells could be detected in samples taken daily during the 12 days survey period, which followed the challenge (Fig. 1). This was established using three different methods: extensive microscopy, QBC and a sensitive PCR assay. In the fourth immunized chimpanzee, Mayoumba, the course of parasitaemia was comparable to that observed in controls.

Five months later, these six chimpanzees underwent a second similar challenge by 20,000 *P. falciparum* sporozoites. Each animal presented the same pattern as that observed following the first challenge, a complete absence of parasites in the three previously protected chimpanzees (Fig. 1) and a blood parasitaemia starting on days six or seven in Mayoumba and in the two control animals. Although the number of animals did not allow results to reach statistical significance, they confirm the parasitological reproducibility of the model, since the status of protection or non-protection was observed upon successive challenges, similarly to those 52 previous challenges reported elsewhere [1]. The consistency upon successive challenges performed after a long delay has in fact greater value than a statistically significant result on a single challenge in a larger group. For instance, 5 consecutive challenges performed over a year in chimpanzees immunized by either optimally irradiated sporozoites or over-irradiated sporozoites, provided in the first group consistent protection and in the second group, consistent parasitemia with exactly the same pattern at each challenge [1]. In contrast to mice, the chimpanzees being rare and precious animals, only limited numbers can be studied but the reproducibility of results upon successive challenges provides in our opinion, greater confidence in the protection data than larger groups of animals challenged once. In addition, if the two challenges were cumulated, the difference of 0 protected out of 4 control challenged vs. 6 out of 8 protected in the immunized group would become significant.

Although LSA-3 protein and peptides formulations have proved able to induce strong antibody and cellular responses in lower as well as higher primates [7], [10], [11], [12] we could not detect any anti-LSA-3 antibodies in the four chimpanzees immunized with LSA-3 DNA, either by ELISA using a large series of 21 peptides and 3 recombinant proteins, or by an immunofluorescence antibody assay on *P.falciparum* sporozoite and Liver stages, throughout the experiment i.e. including after challenge (data not shown). This finding contrasts with results recorded in mice [6] but mimics the results from genetic immunization of humans [13]. The high antibody titers induced in lower primates and in rodents with both the LSA-3 and CS DNA-constructs [6], [14], [15] further underline the discrepancy with regard to genetic immunization between higher primates and other animals.

**Figure 2:**
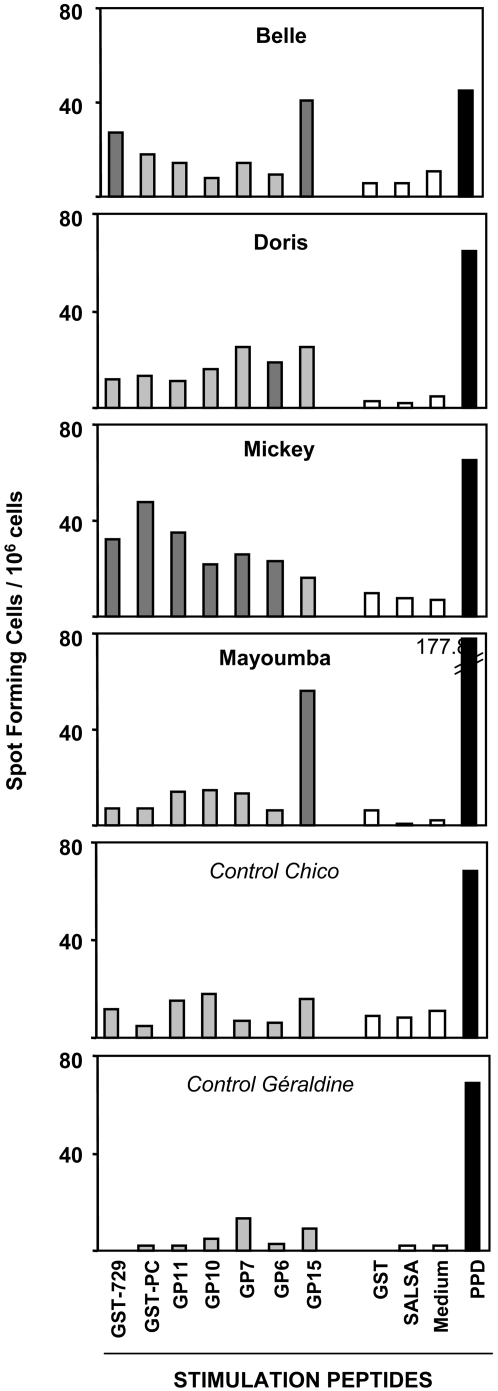
Interferon-gamma ELISpot assays. Bars correspond to the mean number of spot forming cells (SFC) per 10^6^ cells from triplicate wells, following stimulation by LSA-3 peptides (in gray), negative controls (open bars) or positive control PPD (in black) (see details in Mat. and Meth.). Results were considered as positive (dark gray) when SFC were both >20 and >the highest mean SFCs of negative controls plus two S.D. (not shown). Peptides that gave negative results in all assays are not reported here.

**Figure 3:**
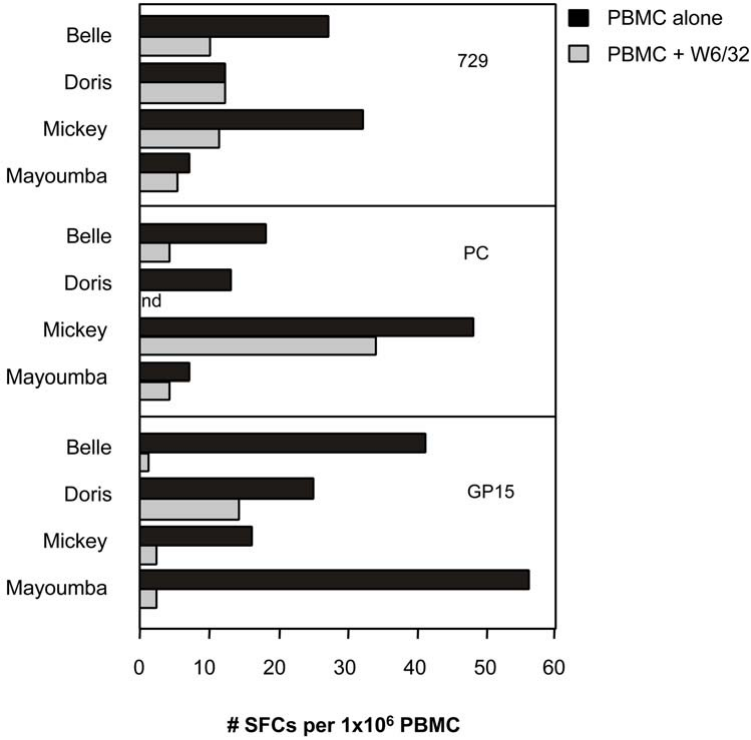
LSA-3 DNA immunization induces specific IFN-γ responses which are inhibited by an anti-MHC Class I blocking antibody. Chimpanzee's PBMC collected at week 30 were assayed for IFN-γ production in an ELISPOT assay with recombinant proteins GST-729 and GST-PC or peptide GP15, either in the absence (solid bars) or in the presence (open bars) of the HLA monomorphic monoclonal antibody W6/32 (Mat. and Meth.). Results are expressed as the mean number of IFN-γ SFCs per 1×10^6^ PBMC. The number of SFCs obtained with PBMC from the two negative control chimpanzees was not significant (not shown).

The central role of interferon-gamma (IFN-γ) in inhibiting liver stage development has been repeatedly stressed under *in vitro* [16], [17] as well as *in vivo* conditions [1], [2], [18], [19], [20], [21]. Our immunization and challenges experiments in mice, *Aotus* monkeys and Chimpanzees, all point to CD4 T-helper1 dependant IFN-γ secretion as the best surrogate of protection. In view of the large number of T-cell epitopes identified within LSA-3, we therefore focused our analysis of cellular responses towards 21 polypeptides spanning the protein employing ELISpot-IFN-γ assays. Positive ELISpot responses were obtained in each of the four LSA-3 immunized chimpanzees (Fig. 2). These responses were specific as negative results were recorded with the three negative controls, and in control animals stimulated *in vitro* with LSA-3. Patterns varied from one animal to the other, as expected for outbred animals. Class I restriction was investigated using the W6/32 pan-specific anti-Class I blocking antibody as an indicator of CTL-type responses [22]. Results, summarized in Figure 3, indicate a mixed Class I and Class II type of response to selected peptides, with the exception of GP15 where responses were almost entirely class I dependent. In the non-protected immunized animal, Mayoumba, T-cell responses were restricted to only peptide GP15, potentially suggesting that responses to other regions of the molecule are critical for protection. In contrast with results obtained in DNA-immunized mice, no response to the R2 repeat region (recombinant protein GST-NN and peptides RE, GP12-13-14) (Fig. 3) was observed. Conversely, the results confirm the existence of T-cell epitopes in the C-terminus unique sequences NR-B and NR-C (Fig. 3) as observed previously in chimpanzees following immunization with polypeptide formulations [1], [6], [7], [10], [12].

The chimpanzee is the primate most closely relative to humans, and the suitability of this model for pre-erythrocytic vaccine evaluation has already been discussed extensively earlier [1], [6], [7], [10], [12]. In the absence of splenectomy the blood stage infection is limited and self –resolving. In contrast the chimpanzee is fully susceptible to sporozoite invasion and liver stages development of *P. falciparum* in terms of numbers obtained and intrahepatic maturation of schizonts, as much as are humans [23]. The short-term blood stage parasitemia is here used only a marker of absence of blockade of the parasite at liver stage, it corresponds to the first invasion of red cells by merozoites released from the intrahepatocytic schizonts, followed by two cycles of intra-erythrocytic schizogony. Moreover, chimpanzees, like humans and in contrast to rodent models, are fully susceptible to successive challenges by sporozoites, showing reproducible emergence of blood stages on each challenge [1], [6], [7], [10], [12]. It is therefore fitted to the assessment of pre-erythrocytic vaccines. LSA3 is only expressed at sporozoite and liver stage, although leaky expression in blood stages has been reported and can indeed be obtained though only under artefactual conditions of cultivation at laboratory level (Prieur E. et al in preparation).

In conclusion, the pre-clinical data presented here reinforces the potential of DNA immunization in humans, which has previously been questioned in view of the small number of immune effectors elicited in this host [13], [24] as compared to animal models [3], [4], [6], [25]. It also confirms the protective potential of the LSA-3 antigen [1].

The results indeed support the use of this delivery system, despite the limited immune responses induced in the chimpanzee, since these were nevertheless sufficient to induce sterile protection in the closest living evolutionary relative to humans. It is noteworthy that the types of immune response which were induced by DNA in chimpanzees, i.e. specific cellular responses but a lack of detectable B-cell responses, are reminiscent of those observed in humans immunized by the same means either by a *P. falciparum* CS-encoding construct expressed by the same vector [13] or using genes from other pathogens such as hepatitis B, or influenza (Sadoff, personal communication). Therefore, these results demonstrate the immunological relevance of the chimpanzee to human beings for pre-clinical vaccine development [18], [19]. In the CS study, the protective status of the volunteers was not investigated in view of the scarcity of immune responses. Our results suggest that even if restricted in number, the immune responses, which were generated by LSA3, were efficient in controlling malaria. A DNA plasmid encoding the *Pf*LSA-3 full-length, including an extreme 3′ region encoding the C-terminus very hydrophobic region, has been previously administered in a Phase IIa trial as part of a DNA formulation comprising a mixture of five genes, without success [26]. However the LSA3 construct employed in this experiment had, in contrast to ours which excluded the region coding for the extreme C-terminus, an extremely low level of expression both in mice and in humans and induced very scarce if any immune response in a very limited number of volunteers. Therefore this experiment does not rule out the potential of LSA3 genetic immunisation in humans.

A critical aspect of an effective malaria vaccine is boosting and immunological memory. In contrast with many other malaria vaccination experiments, we deliberately performed two successive sporozoite challenges and these only after a long delay following the last immunization (3.5 and 9 months). Our results demonstrated that immunity induced by LSA-3 DNA vaccination persists for a long duration without the necessity of an artificial boost [27], [28].

These results are in keeping with previous reports showing significant levels of protection in large mammals following DNA vaccination against schistosoma, in the absence of a detectable immune response [29]. This suggests that the true potential of genetic immunisation might have been drastically underestimated based on assessments of overall immunogenicity in humans using vaccines against diseases where challenges are either hardly feasible or not customary (e.g. HIV, HBV, HCV, TB, Influenza). The lack of parallels with more classical immunisation methods might also be the reason behind such hasty conclusions being made, despite the fact that mechanisms of defence are in fact notoriously poorly understood, or have yet to be identified, for numerous pathogens. The situation is further complicated by circumstances where very promising data is obtained in mice following DNA vaccination, while in humans very poor immune responses with high inter-individual variability are subsequently recorded [30].

Genetic immunisation is a very unusual means of immunisation and it may therefore not be surprising that it can generate unusual patterns of immune responses that differ from those obtained with proteins or live vectors. For instance, DNA might preferentially induce a subset of immune competent cells which are either poorly characterized today, not detectable by current assays developed for investigating other immunisation methods, or present in limited numbers in peripheral blood samples. The results obtained in our study using an antigen which has proved effective using more classical antigen delivery systems in the same host, indicate that genetic immunisation by LSA3 now deserves to be investigated in clinical trials. Indeed, these findings suggest that the relative scarcity of an immune response should not necessarily exclude the assessment of the protective efficacy of a vaccine candidate when ethically feasible.

## Materials and Methods

### DNA construct

The LSA-3 plasmid vaccine, denoted as VR-LSA3, was constructed by cloning a PCR fragment corresponding to almost the entire *lsa-3* gene of the *P. falciparum* clone 3D7, except for the extreme 3′ end encoding a highly hydrophobic region, as an in-frame fusion with the human Tissue Plasminogen Activator leader sequence encoded upstream of the *Bam*HI site of plasmid pVR1020 (Fig. 1). In the *lsa-3* gene from reference strain K1 [1] and EMBL accession number AJ007010), primers pVR20.5′A (nucl. 432–460) and pVR.3′B (nucl. 5095–5064) amplify a 4663 bp fragment, whereas in the vaccine VR-LSA3, the lsa-3 insert, which was amplified from clone 3D7 DNA [31] and EMBL accession number AE001424) using these primers, spans over 3920 due to a shorter repeat region R2 in 3D7 compared to K1 parasites (data not shown). Following complete sequence verification of the VR-LSA3 insert, large quantities of super coiled plasmids were first prepared from a transformed E.coli DH5α strain cultured in a 20 liter bioreactor under optimal conditions of oxygenation and nutrition (in order to minimize the formation of relaxed plasmid DNA), and further purified using EndoFree Plasmid Giga Kits (Qiagen, Germany). The DNA solution was adjusted to 1 mg/ml using endotoxin-free PBS buffer, aliquoted in 1 ml samples and stored at −20°C. Endotoxin was present at concentrations between 5 and 50 E.U./mg DNA, as determined with the *Limulus Amebocyte Lysate* test (BioWhittaker, USA). Efficient expression of LSA-3 by VR-LSA3 was verified: 1) through *in vitro* cultures of transfected CHO cells (Lipofectamine, Gibco-BRL, USA) by immunofluorescence using anti-GST-729 and anti-GST-PC antibodies [1], directed against the N- and C-terminal part of the antigen, respectively (data not shown), 2) *in vivo* by intramuscular injections of the plasmid in mice followed by immune response analysis [6]. Control plasmid VR2402 was prepared in the same manner as plasmid VR-LSA3 and consisted of the VR1020 vector expressing a non-relevant antigen, the Respiratory Syncytial Virus protein (C. de Taisne, Aventis-Pasteur, personal communication).

### Animals and immunization

Six adult chimpanzees (*Pan troglodytes*) with no previous exposure to malaria were included in the study.

Animals were handled in accordance with standard operating procedures in the Centre International de Recherches Médicales de Franceville (CIRMF). The protocol of the present study was discussed, revised and approved by the CIRMF Committee of Ethic and Control of Animal Use N°12, which include a physician, three veterinaries specialized in primates, one being an ethologist, a reknown primatologist, a scientist, a representative of the Ministry of foreign affairs, and the Director of the CIRMF also a scientist.

For each animal, a 1 mg dose of the adequate plasmid solubilized in 2 ml of endototoxin-free PBS was split and injected intramuscularly in 2 symmetrical sites (tibialis anterior) each time at weeks 0, 4 and 9.

### Challenge and parasitemia follow-up

At weeks 24 and 49, all animals were challenged intravenously with 20,000 sporozoites (NF54 strain, the parasite strain used for challenge of human volunteers) obtained from dissected salivary glands of infected female mosquitoes (*Anopheles gambiae*, the mosquitoes species dominant in African settings) as previously described, reared in Nijmegen under strict containment conditions [32]. For protection assessment, blood samples were collected daily from days 4 to 12 post-challenge and blood parasitaemias were determined by extensive examination of Giemsa-stained thick and thin film by experienced microscopists, by QBC and by PCR as previously described [33], [34]. Following the observation period, all animals were curatively treated with chloroquine, irrespective of their protective status.

### Immune responses

Plasma antibody responses were measured by standard ELISA and IFAT. For ELISA, coated antigens correspond to the LSA-3 recombinant proteins and synthetic peptides described below. IFAT were performed on wet or dry NF54 sporozoites and day six post-challenge liver stages from a chimpanzee as previously described [1].

T-cell responses were evaluated on PBLs. Forty ml of blood were collected on heparin thirty weeks after the first immunization. The number of IFN-γ- producing cells was determined in freshly Ficoll-isolated, unstimulated PBLs 40 hr after incubation with the indicated antigens. Nitrocellulose-backed microtiter plates (MAHAS45; Millipore Corp., Molsheim, France) were coated with 50 µl of a 5 µg/ml solution of an anti-human IFN-γ capture antibody (clone 350B10G6, Biosource International, California, USA) diluted in carbonate buffer 50 mM, pH 9.6. After overnight incubation at +4°C, the wells were washed three times with PBS, and were incubated with PBS containing 5% human AB serum (Institute Jacques Boy S.A., Reims, France). The plates were washed again three times with PBS. PBL suspensions at 5×10^5^ cells/well in 200 µl RPMI 160 medium supplemented with Penicillin-streptomycin, L-glutamine and Hepes (all purchased from Gibco BRL, Life Technologies, Rockville, USA) and with 10% human AB serum, together with 10 µg/ml of either recombinant protein or synthetic peptide were introduced in each well. In some assays, a 10 µg/ml solution of the blocking antibody W6/32 (American Type Culture Collection, Rockville, MD) to a monomorphic determinant from HLA class I molecules was introduced in the wells. Plates were then incubated for 40 hr at 37°C in a humidified 5% CO2 air incubator. The plates were then washed three times with PBS containing 0.05% Tween 20 (PBST), and three times with PBS only. Wells were then overlaid with 50 µl of a solution of 2.5 µg/ml biotinylated anti-human IFN-γ detection antibody (clone 67F12A8, Biosource International, California, USA) in PBS 3 hr at room temperature. Plates were washed three times with PBS/T, and three times with PBS before being treated with 50 µl/well of peroxydase-labelled streptavidin (Boehringer Mannheim GmbH, Germany) at a dilution of 1/2000 in PBS. After one-hour incubation, plates were washed extensively with PBS/T and PBS. Spots were revealed with the BCIP/NBT reagent set (Promega, Madison Wi, USA) at sites where individual cells had produced IFN-γ. Results are expressed as the number of spot forming cells (SFCs) per 10^6^ PBL.

PBL were stimulated with: 1) LSA-3 recombinant proteins GST-729, GST-NN and GST-PC, which have been previously described [1] and were designed so as to cover 95% of the LSA-3 antigen; 2) The LSA-3 synthetic peptides previously reported in ref. 17 and which are located in the K1 reference strain as follow: GP2 (aa44-119), GP1 (aa100-222), GP14 (aa501-596), GP13 (aa646-706), GP12 (aa692-854), GP11 (aa840-907), GP10 (aa893-999), GP9 (aa985-1040), GP8 (1026-1095), GP7 (aa1081-1157), GP6 (aa1143-1255), GP5 (aa1241-1346), GP4 (aa1332-1517), GP3 (aa1503-1575) and GP15 (aa1601-1712); 3) Negative controls, which correspond to the culture medium alone, the non-relevant SALSA1 peptide [35] or the glutathion-S-transferase from *Schistosoma mansoni* expressed from the pGEX vector (Invitrogen, The Netherlands); 4) the positive control Purified Protein Derivative (PPD).
